# Bactericidal and Anti-Biofilm Activity of the FtsZ Inhibitor C109 against *Acinetobacter baumannii*

**DOI:** 10.3390/antibiotics11111571

**Published:** 2022-11-08

**Authors:** Viola Camilla Scoffone, Samuele Irudal, Aseel AbuAlshaar, Aurora Piazza, Gabriele Trespidi, Giulia Barbieri, Vadim Makarov, Roberta Migliavacca, Edda De Rossi, Silvia Buroni

**Affiliations:** 1 Department of Biology and Biotechnology “Lazzaro Spallanzani”, University of Pavia, 27100 Pavia, Italy; 2 Unit of Microbiology and Clinical Microbiology, Department of Clinical-Surgical, Diagnostic and Pediatric Sciences, University of Pavia, 27100 Pavia, Italy; 3 Research Center of Biotechnology RAS, 119071 Moscow, Russia

**Keywords:** *Acinetobacter baumannii*, drug resistance, biofilm, FtsZ

## Abstract

In the last few years, *Acinetobacter baumannii* has ranked as a number one priority due to its Multi Drug Resistant phenotype. The different metabolic states, such as the one adopted when growing as biofilm, help the bacterium to resist a wide variety of compounds, placing the discovery of new molecules able to counteract this pathogen as a topic of utmost importance. In this context, bacterial cell division machinery and the conserved protein FtsZ are considered very interesting cellular targets. The benzothiadiazole compound C109 is able to inhibit bacterial growth and to block FtsZ GTPase and polymerization activities in *Burkholderia cenocepacia*, *Pseudomonas aeruginosa*, and *Staphylococcus aureus*. In this work, the activity of C109 was tested against a panel of antibiotic sensitive and resistant *A. baumannii* strains. Its ability to inhibit biofilm formation was explored, together with its activity against the *A. baumannii* FtsZ purified protein. Our results indicated that C109 has good MIC values against *A. baumannii* clinical isolates. Moreover, its antibiofilm activity makes it an interesting alternative treatment, effective against diverse metabolic states. Finally, its activity was confirmed against *A. baumannii* FtsZ.

## 1. Introduction

*Acinetobacter baumannii* is raising concern due to its extensive drug resistance and high mortality rate [1], especially for Intensive Care Units patients [2,3]. This Gram-negative bacterium, belonging to the ESKAPE group, usually causes ventilator-associated pneumonia [4] and infections involving skin and soft tissue, urinary tract, and bloodstream [1]. Its ability to acquire plasmids, transposons and integrons facilitates high adaptability, and the diffusion of resistance genes [5]. The increase in the use of beta-lactams has contributed to the spread of Multi Drug Resistant (MDR) strains, the prevalence of which is estimated to be 79.9% in pneumonia patients [5], requiring treatment with the last-resort antibiotic carbapenem. However, the emergence of carbapenem resistant-*A. baumannii* (CR-Ab) poses an additional threat [6]. Indeed, in 2018 CR-Ab ranked as the number one priority, according to the WHO, due to carbapenem resistance being associated with a broad range of co-resistance against other classes of antibiotics [7]. A different alternative is now provided by the novel cefiderocol cephalosporin, which showed promising activity against ESKAPE and *A. baumannii* isolates. A high percentage of unstable heteroresistence still arose after monotherapy treatment, highlighting how important combination therapy is [8]. Conversely, high loading dose colistin monotherapy successfully eradicated MDR-*A. baumannii* infections [9].

Along with its virulence factors, such as outer membrane proteins, capsular polysaccharides, and iron acquisition systems [10], the genetic array of this pathogen allows its survival and diffusion in a hostile environment, such as clinical settings [11]. Therefore, research is nowadays focusing on alternative treatments which could dampen antimicrobial resistance by decreasing antibiotic assumption. Bacteriophages are harmless to humans and a strain-specific weapon, capable of precisely hijacking bacterial metabolism, leading to cell lysis [12,13,14]. Still, concern arises about the possibility of interbacterial DNA transfer by phage diffusion and the rise of phage-resistant strains [15]. To note, the phage-resistant phenotype is associated with a decrease in antibiotic resistance when combined treatments are performed [14]. Bacteriocins, a class of Antimicrobial Peptides (AMPs), are host-produced peptides possessing bactericidal and immunomodulatory properties [16]. Recent interest has emerged in these compounds, as they show a low tendency to select resistant strains, broad-spectrum activity, rapid killing action, and high clinical efficacy against different MDR strains [17,18,19,20]. These properties result from their membrane disruptive capacity or by their ability to hit internal targets [16]. Unfortunately, their clinical application is often limited by side effects, including hemolytic activity, immunogenicity, and drug resistance after long-term use [21]. However, both bacteriophages and AMPs can be used in combination with antibiotics, enhancing their potency and even decreasing their administration dose [22,23]. Another issue directly related to the metabolic state of the bacterium is its ability to form biofilms, which are noteworthy as being less susceptible to the effects of standard treatments.

**Figure 1:**
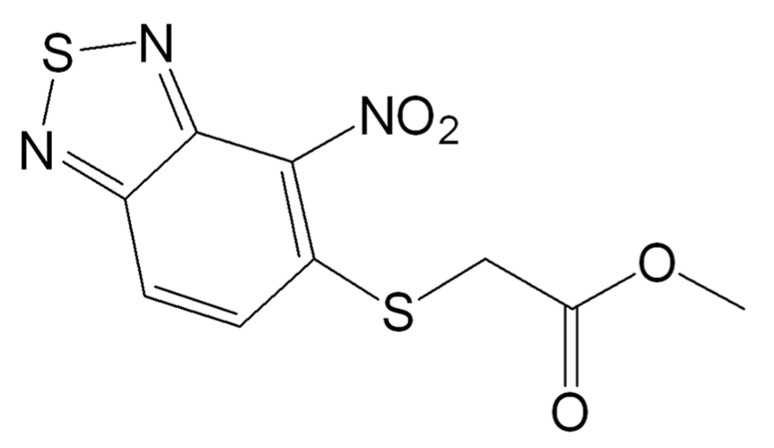
Chemical structure of C109 compound.

In this light, bacterial division machinery has acquired more and more importance [24,25,26], since it could represent the ideal target for the development of new compounds effective against MDR bacteria. Several molecules have been found to interfere with the divisome, mainly targeting the conserved protein FtsZ, “Filamenting temperature-sensitive mutant Z”, involved in the midpoint Z ring formation needed for cell division [27,28]. In the last few years, a broad-spectrum compound, methyl [(4-nitro-2,1,3-benzothiadiazol-5-yl)thio]acetate, named C109 (Figure 1), has surprised researchers with its ability to inhibit purified FtsZ, preventing both its GTPase and polymerization activities and being active at low concentrations against different Gram-negative and Gram-positive bacteria, including *Burkholderia cenocepacia*, *Pseudomonas aeruginosa* and *Staphylococcus aureus* [29,30,31,32,33].

Considering the urgent need for new treatment against *A. baumannii*, the effect of C109 against sensitive and resistant strains was explored, together with its biofilm inhibitory activity. Finally, *A. baumannii* FtsZ was expressed and purified to evaluate GTPase and polymerization inhibition in the presence of the compound.

## 2. Results

### 2.1. Activity of C109 against Sensitive and Resistant A. baumannii Strains

The activity of C109 was tested against a total of 108 *A. baumannii* strains by means of the microdilution method in Mueller Hinton (MH) broth. The bacterial load was always between 3.1 × 10^3^ and 2.1 × 10^5^ CFU/ml. The minimal inhibitory concentration (MIC) of C109 against the reference strain *A. baumannii* ATCC19606 was 8–16 mg/L and 8–32 mg/L for the clinical strain, while the minimal bactericidal concentration (MBC) was 16 mg/L.

**Figure 2:**
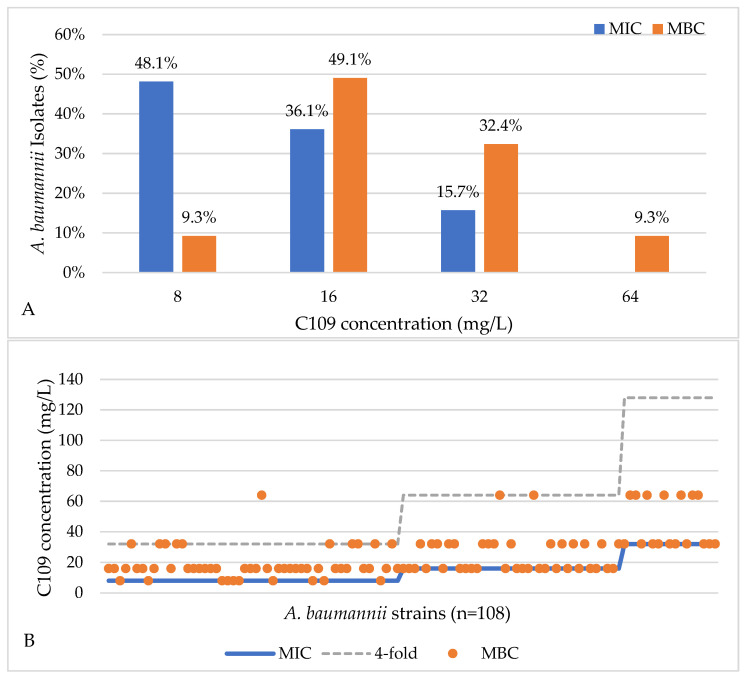
(**A**) MIC and MBC values of C109 obtained for *A. baumannii* strains (n = 108). (**B**) the bactericidal effect of C109 on *A. baumannii* isolates (n = 107/108).

Among the 108 clinical isolates, *A. baumannii* strains 52 (48.1%), 39 (36.1%), and 17 (15.7%) displayed MIC values of 8, 16, and 32 mg/L, respectively (Figure 2A). These results showed that the most representative MIC values among clinical isolates were equal to, or lower than, those of the reference strains, i.e., 16 mg/L. The MBCs for the same *A. baumannii* (n = 108) strains were 8, 16, 32, and 64 mg/L for 10 (9.3%), 53 (49.1%), 35 (32.4%), and 10 (9.3%) strains, respectively (Figure 2A), demonstrating that 16 mg/L, the most represented value for the MBC, was the same for the reference strains. In fact, C109 showed a bactericidal effect in 99% (n = 107/108) of the cases, with MBC being not more than fourfold higher than the MIC (Figure 2B). C109 did not show the bactericidal effect on one isolate (1%).

### 2.2. C109 Biofilm Inhibitory Activity against A. baumannii Strains

To test the biofilm inhibitory acitivity of C109, *A. baumannii* ATCC19606 and three strains, not clonally related by Pulsed-Field Gel Electrophoresis (PFGE) and Multi-Locus Sequence Typing (MLST), were selected as representatives for all the strains. *A. baumannii* ATCC19606 belongs to the ST52 group, MO5 belongs to ST78, ST for HU5 is unkown, while the strain 560380 belongs to the ST2.

**Figure 3:**
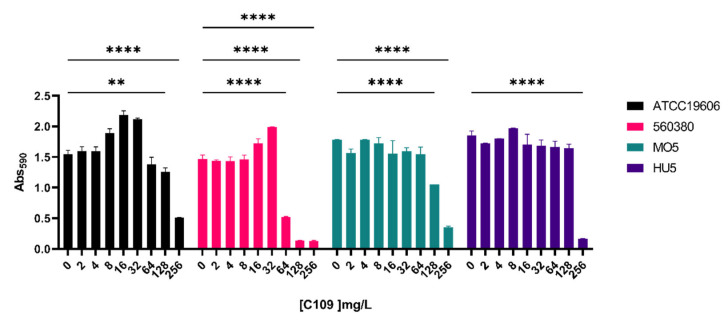
Effect of increasing concentration of C109 against biofilm formation ability of *A. baumannii* strains: ATCC19606 (**in black**), 560380 (**in pink**), MO5 (**in green**) and HU5 (**in purple**). The C109 concentration is indicated below each bar in mg/L. Mean ± standard error, n = 2. ** *p* < 0.01; **** *p* < 0.0001 (two-way ANOVA test).

The biofilm inhibitory activity of the compound C109 against *A. baumannii* strains was first determined using a 96-well microplate crystal violet assay [34]. C109 was tested at increasing concentrations, ranging from 2 to 256 mg/L. Under the tested conditions, the biofilm formation ability of the *A. baumannii* ATCC19606 and MO5 strains significantly decreased in the presence of 128 mg/L of C109, while that of the *A. baumannii* HU5 strain significantly reduced only in the presence of 256 mg/L of C109. The biofilm of the 560380 strain was already significantly altered in the presence of 64 mg/L of the compound (Figure 3).

**Figure 4:**
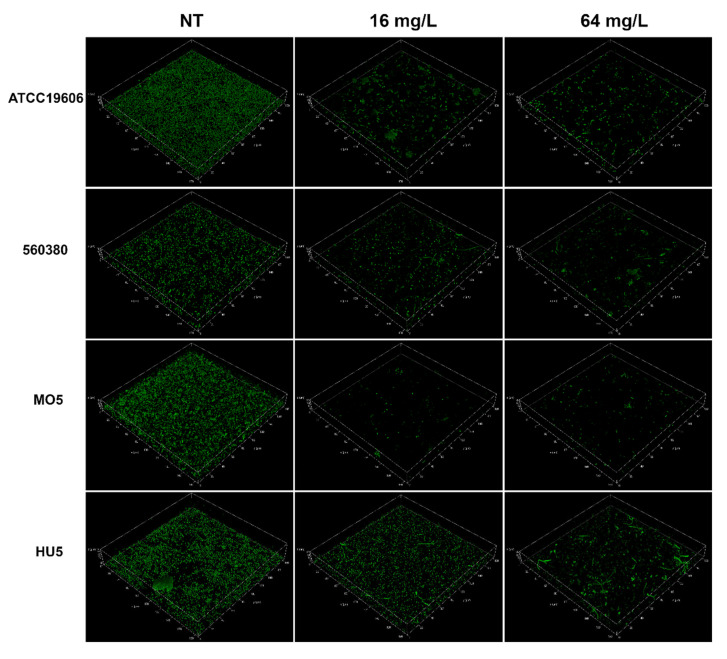
CLSM images of *A. baumannii* biofilms grown in four-well chambered coverslips. Pictures were taken with an overall magnification of 400×. Cells were grown overnight at 37 °C in MH with no C109 (NT), 16 mg/L of C109 or 64 mg/L of C109. Seventy planes at equal distances along the *Z*-axis of the biofilm were imaged by CLSM. 2D images were stacked to reconstruct the 3D biofilm image. Scale bar represents 28 μm.

Confocal laser scanning microscopy (CLSM) analysis was used to better characterize the effect of the C109 on *A. baumannii* biofilm morphology. *A. baumannii* strains ATCC196060, 560380, MO5 and HU5 were grown as static cultures in four-well chambered coverslips in the absence, or in the presence, of different concentrations of C109 (16, 64 and 256 mg/L) at 37 °C. Biofilms were stained with Syto 9, staining nucleic acids of all cells. Representative 3D image reconstructions of biofilms formed by each strain in the presence of 0 (untreated), 16 and 64 mg/L of C109 are reported in Figure 4. The images show that, from a qualitative point of view, the biofilm produced by the *A. baumannii* strains ATCC19606, 560380 and MO5 was already reduced at 16 mg/L of C109. In the case of the strain HU5, the same effect was observed only at 64 mg/L, in accordance with its lower susceptibility to the compound (Figure 3).

**Figure 5:**
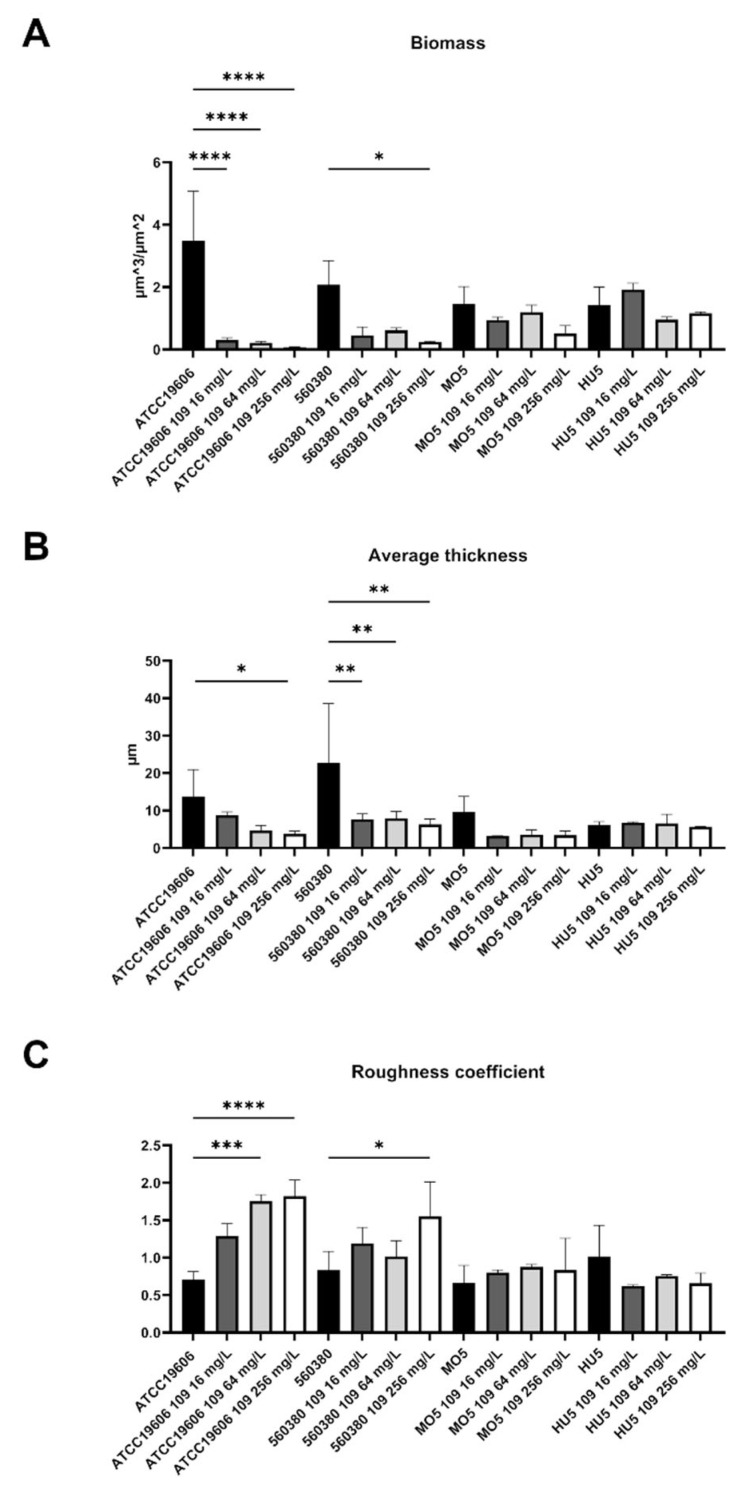
Analysis of biofilm properties by COMSTAT 2. (**A**) Measurements of total biomass, (**B**) average thickness, and (**C**) roughness coefficient. Data are the mean ± SD of the results from three independent replicates. * *p* < 0.1, ** *p* < 0.01, *** *p* < 0.001, **** *p* < 0.0001 (two-way ANOVA test).

A specific biofilm morphology analysis was carried out with COMSTAT 2. These analyses showed that the biomass of the biofilm produced by ATCC19606 had already decreased significantly in the presence of 16 mg/L C109 (Figure 5A), while that of the strain 560380 decreased significantly only in the presence of 256 mg/L of C109 (Figure 5A). The biomass of the biofilm formed by the MO5 strain showed only a slight decrease in the presence of 256 mg/L of C109 (Figure 5A). On the other hand, the biomass of the biofilm produced by the HU5 strain was not altered in the presence of C109 at the tested concentrations (Figure 5A). However, the last two strains had the ability to form a biomass which was lower in respect to the first two.

The average thickness of the biofilm produced by the *A. baumannii* ATCC19606 significantly decreased in the presence of increasing concentrations of C109 (Figure 5B). At the same time, the average thickness of the biofilm 560380 had already significantly decreased in the presence of 16 mg/L of C109 (Figure 5B). The average thickness of the biofilm formed by the strain MO5 showed a decrease, which was not statistically significant (Figure 5B). The C109 treatment did not affect the average thickness of the biofilms formed by the strains HU5 (Figure 5B). In this case, the thickness of the biofilm formed by MO5 and HU5 was lower compared to the ATCC19606 and 560380 strains.

Using COMSTAT, the roughness coefficient (variation in thickness calculated from the distribution of the biofilm thickness) was also analyzed, which provided an indicator of biofilm heterogeneity. The data showed that, in the case of the ATCC19606 strain, the C109 treatment induced an increase in the roughness coefficient, suggesting that the biofilm was less homogeneous (Figure 5C). The roughness of the biofilm formed by the strain 560380 increased significantly only in the presence of 256 mg/L of C109 (Figure 5C). In the case of the two clinical isolates, MO5 and HU5, the roughness coefficient of the biofilms was not affected by the compound C109 (Figure 5C).

**Figure 6:**
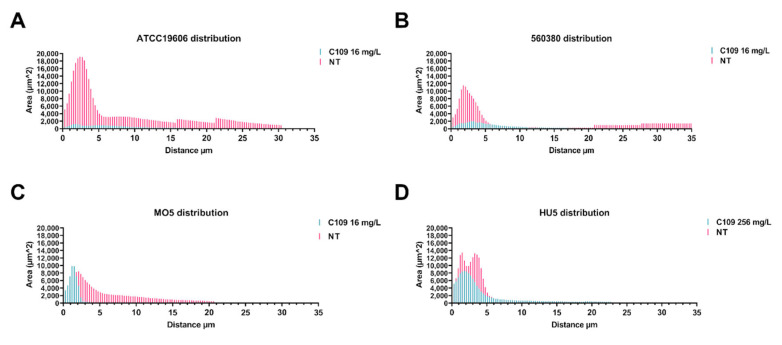
Analysis of biofilm properties by COMSTAT 2% of the area occupied by biofilm distribution, (**A**) ATCC19606, (**B**) 560380, (**C**) MO5 and (**D**) HU5. Data are the mean ± SD of the results from three independent replicates.

The biofilm distribution on the *Z*-axis of the treated samples changed drastically in the strains ATCC19606 and 560380, while the biofilm distribution of the strain MO5 was less affected by the treatment with 16 mg/L C109 (Figure 6A–C). On the other hand, the biofilm of the HU5 strain was only partially altered in the presence of 256 mg/L C109 (Figure 6D).

### 2.3. C109 Activity on Purified A. baumannii FtsZ

**Figure 7:**
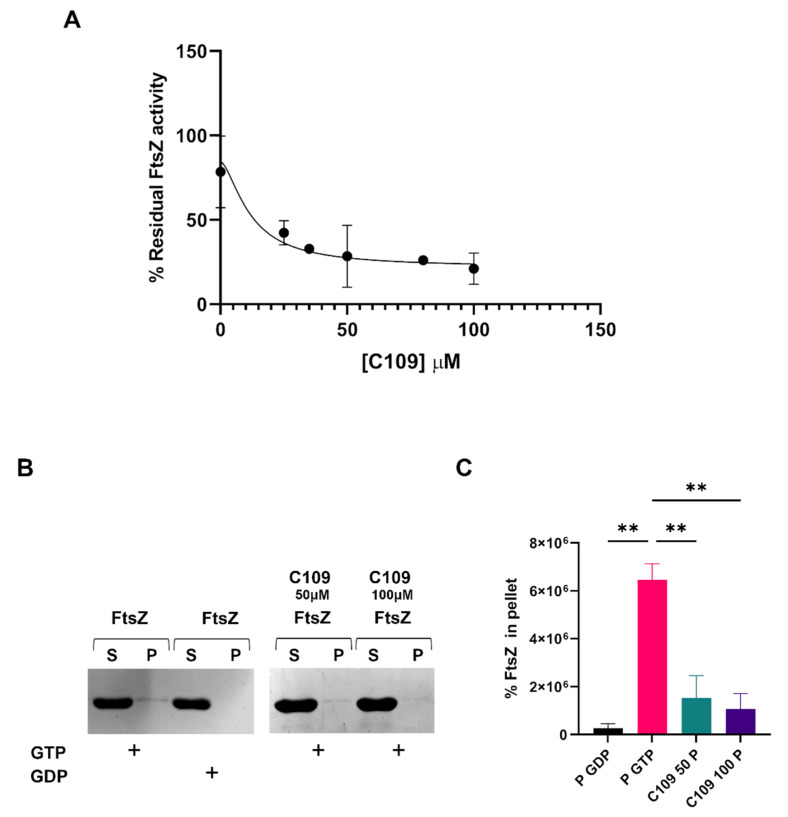
GTPase activity and polymerization assays on *A. baumannii* FtsZ in the presence of C109. (**A**) IC_50_ of C109 against *A. baumannii* FtsZ. (**B**) Sedimentation assay of FtsZ in the presence, or absence, of C109, GTP and GDP. P, insoluble fraction (pellet); S, soluble fraction (supernatant). (**C**) Relative quantification of FtsZ percentage in the pellet of the indicated samples obtained by densitometry analysis. Data are the mean ± SD of the results from three different replicates; images are representative of at least three different experiments. ** *p* < 0.01 (one-way ANOVA test).

FtsZ is the cellular target of the compound C109 [30]. C109 is able to block the GTPase activity and the polymerization of FtsZ in *B. cenocepacia* and *P. aeruginosa*, while in *S. aureus* it blocks only the GTPase activity [31,33]. To evaluate its activity against the *A. baumannii* FtsZ, the protein was expressed and purified, as described in the Materials and Methods Section 4.5. The GTPase activity of the recombinant FtsZ of *A. baumannii* ATCC19606 was evaluated, as previously described [30], with minor modifications. The achieved results showed that C109 inhibited the FtsZ GTPase activity with a 50% inhibitory concentration (IC_50_) of 11.84 μM (Figure 7A), which indicated that the GTPase activity of FtsZ was impaired by the compound C109. In order to understand if C109 was also able to block FtsZ polymerization in *A. baumannii*, an in vitro sedimentation assay was performed [30]. *A. baumannii* FtsZ polimerized in vitro in the presence of GTP (its substrate) and not in the presence of GDP (its product). When C109 was added to the reaction at increasing concentration (50 and 100 μM), the polymerization was already blocked at the lowest concentration used (Figure 7B,C).

These results demonstrated that the compound C109 blocked both the GTPase and the polymerization activity of FtsZ of *A. baumannii*, as previously described regarding *B. cenocepacia* and *P. aeruginosa* [30,31].

## 3. Discussion

Recently, the WHO has recommended a global strategy to decrease the appearance of antibiotic resistant isolates and to identify new treatments. The development of new compounds active against Carbapenem-resistant *A. baumannii* is a priority. *A. baumannii* is the causative agent of 2% of healthcare-associated infections [35]. In particular, the most frequent infections with the highest mortality rates are ventilator-associated pneumonia and bacteremia, identified in patients with prolonged periods of hospitalization [36]. Of note, *A. baumannii* has been detected in sputum cultures and tracheal aspirates from COVID-19 mechanically ventilated patients [37].

The ability of this bacterium to persist on abiotic surfaces and to resist hostile environments, such as clinical settings, make it a dangerous pathogen. Therefore, focusing attention on alternative treatments is extremely relevant. In this context, weapons that hit the bacterium during different metabolic states are of the most importance. It is noteworthy that bacteria growing as biofilm show different susceptibility to antibiotics, due to their metabolism being different with respect to planktonic cells. Hence, bacterial cell division machinery and the conserved protein FtsZ are considered very interesting cellular targets.

The benzothiadiazole derivative C109 inhibits FtsZ GTPase and polymerization activities in *B. cenocepacia* and *P. aeruginosa*, while only affecting the GTPase activity in *S. aureus* FtsZ. To face the problem of drug resistance in *A. baumannii*, the activity of C109 was tested against a panel of sensitive and resistant strains. The results showed that C109 MIC and MBC against the *A. baumannii* ATCC19606 reference strain ranged between 8 and 16 mg/L. So, 107 clinical isolates were tested and the most representative MIC and MBC values were 8 and 16 mg/L. The compound C109 showed a bactericidal effect in 99% of the cases. The obtained MIC and MBC values were considered good values, also taking into account the high number of resistant clinical isolates identified [7].

Moreover, in *A. baumannii*, the ability to form biofilm facilitates tolerance toward external stress, desiccation and antimicrobials [38]. The C109 biofilm inhibitory activity was assessed against ATCC19606 and the clinical strains 560380 (MIC = 16 mg/L), MO5 (MIC = 32 mg/L) and HU5 (MIC = 16 mg/L). The results showed that a concentration of C109 four-fold higher than the MIC decreased biofilm formation of the ATCC19606 and MO5 strains, while for the 560380 strain a concentration 2-fold higher than the MIC was sufficient. On the contrary, for the HU5 strain, a concentration of 8-fold the MIC was required to inhibit biofilm formation. To better characterize the biofilm formed by these strains in the presence of the compound C109, a CLSM analysis was performed, revealing that, for the strains ATCC19606, 560380 and MO5, the biofilm was already altered at a low concentration of C109 (16 mg/L), while, as previously shown by the other experiment, the HU5 was less sensitive.

The biofilm morphology was studied using the COMSTAT software and the analyses confirmed the previous results: the ATCC19606 reference strain and the two clinical isolates 560380 and MO5 were sensitive to the compound C109, since the biomass, the average thickness, the roughness and the distribution of the biofilms were perturbed. The clinical isolate MO5 was less sensitive and a higher dose of C109 was required to show biofilm alteration (256 mg/L).

These results are interesting since C109 treatment was shown to also alter biofilm formation in clinical isolates. Considering the main problem of biofilm formation in the nosocomial infections caused by *A. baumannii*, these findings are very promising, establishing C109 as an effective compound at different metabolic states of the bacteria. Moreover, its combination with currently used therapies could further increase its efficacy.

C109 activity was also fully characterized from an in vitro perspective. The cellular target of this compound was identified as the protein FtsZ in Gram-negative and -positive bacteria, *B. cenocepacia*, *P. aeruginosa* and *S. aureus* [30,31,33]. The GTPase activity of *A. baumannii* FtsZ was indeed inhibited by the compound, showing an IC_50_ of 11.84 μM. This value was good, if compared to the previously described compounds derived from cinnamaldehyde [39]. Moreover, C109 also blocked FtsZ polymerization in *B. cenocepacia* and in *P. aeruginosa*. Further structural studies could help to better elucidate C109 molecular interaction with FtsZ. The data presented here demonstrated that C109 interacted with the *A. baumannii* FtsZ inhibiting both functions: the GTPase activity and the polymerization.

In conclusion, our results showed that C109 is one of the first FtsZ inhibitors described as active against the Gram-negative bacterium *A. baumannii* and with good MIC values against clinical isolates. The compound is also characterized by an interesting antibiofilm activity making it an interesting alternative treatment, effective against diverse metabolic states.

## 4. Materials and Methods

### 4.1. Bacterial Strains and Culture Media

*Acinetobacter baumannii* ATCC19606 and 107 *A. baumannii* isolates strains were used. Bacteria were grown aerobically in Mueller-Hinton (MH, Difco, BD, Franklin Lakes, NJ, USA) or Luria-Bertani (LB, Difco, BD, Franklin Lakes, NJ, USA) broth at 37 °C at 200 rpm. *Escherichia coli* BL21(DE3) strain (laboratory collection) was grown in LBbroth at 37 °C with shaking or on LB agar plates and used for recombinant protein expression. Kanamycin (PanReac, AppliChem, ITW Reagent, Glenview, IL, USA) was used at 50 mg/L for plasmid selection and maintenance.

### 4.2. A. baumannii Clinical Isolates Minimum Inhibitory Concentration (MIC) and Minimum Bactericidal Concentration (MBC) Determination

Minimum inhibitory concentrations (MICs) were determined in quadruplicates for a total of 107 *A. baumannii* clinical isolates and *A. baumannii* ATCC19606, using the broth microdilution method, according to EUCAST guidelines, in Muller–Hinton (MH, Difco, BD, Franklin Lakes, NJ, USA) broth [40]. C109 was dissolved in pure DMSO (≥99.9%). Two-fold serial dilutions of C109 in concentrations ranging from 64 to 0.125 mg/L, with a final inoculum of 5 × 10^5^ CFU/mL, were dispensed in each well of the 96-well culture plate. After incubation for 24 h at 35 °C, 30 µL of 0.015% resazurin (Merck, Readington, NJ, USA) was added to all wells, and the culture was further incubated for 2–4 h for the observation of blue to pink color change, indicating bacterial growth [41]. MICs were determined from visual reading before and after adding resazurin as the lowest concentration able to inhibit microbial growth. All experiments were performed by two workers in quadruplicate, and either the *A. baumannii* ATCC19606 strain or clinical strain *A. baumannii* 15C32 were included in each plate as control strains.

Viable colony counts were performed in duplicates for each strain by diluting 2.5 µL from the growth-control well, after inoculation, in 50 µL of sterile distilled water and spreading on MH agar. Plate count was made by counting the colony-forming units (CFUs) after incubation for 24 h at 35 °C. The minimum bactericidal concentrations (MBCs) of C109 were determined for all *A. baumannii* strains (n = 108) by subculturing 10 µL of bacterial culture from wells, corresponding to the MIC value, and two concentrations higher than the MIC value, on MH agar plates. After 24 h of incubation in aerobic conditions at 35 °C, the lowest concentration of C109 that yielded no visible bacterial growth on MH agar plates was recorded as the MBC value [42].

### 4.3. In Vitro Biofilm Inhibition Test in 96-Well Microtiter Plates

Biofilm formation inhibitory activity of C109 compound was tested on *A. baumannii* ATCC19606, 560380, MO5 and HU5 strains using the crystal violet staining method [34]. Bacterial cultures grown for 18 h in MH broth were diluted to OD600 of 0.02 in MH and incubated in 96-well microtiter plates, either in the absence or presence of different concentrations of C109 (2–256 mg/L). After 3 hours of incubation, the supernatant (containing nonadherent cells) was removed and 200 μL of fresh sterile medium (with the same C109 concentrations) was added to each well and incubated for 20 h at 37 °C. Biofilms were quantified by staining with crystal violet. Briefly, planktonic cells were removed, attached cells were washed with sterile saline solution, biofilms were treated with 90% methanol for 15 min, methanol was removed and the plates were air-dried. Plates were stained with 150 μL of 0.1% crystal violet for 20 min. Wells were gently washed twice with water and the surface-associated dye was dissolved in 150 μL of 33% acetic acid. The absorbance measurements at OD600nm were measured in the Glomax (Promega Madison, WI, USA) plate reader.

### 4.4. Biofilm Evaluation by Confocal Laser Scanning Microscopy

Bacteria were cultured O/N in MH and diluted to an OD600 = 0.01 in the same medium. Bacteria were incubated in a four-well chambered coverslip μ-Slide (Ibidi) at 37 °C, in the presence of different concentrations of C109 (16, 64 and 256 mg/L). The compound C109 was dissolved in pure DMSO (≥99.9%), and the volume of C109 added was always 1/200 of the final volume, since this amount of DMSO was shown not to affect biofilm formation. After three hours of incubation the medium was removed, along with nonadherent cells, and fresh medium containing the same C109 concentrations, was added to the chambers. After over-night incubation, the medium was removed, and biofilms were washed with PBS 1X and stained with Syto 9 (Invitrogen, Waltham, MA, USA) at the final concentration of 5 μM. A 63X oil immersion objective Leica DMi8 with 500- to 530-nm (green fluorescence representing Syto 9) emission filters were used to take five snapshots randomly at different positions in the confocal field of each chamber. The Z-slices were obtained every 0.3 microns. For visualization and processing of biofilm images, ImageJ was used. The thickness, biomass, roughness coefficient, and biofilm distribution were measured using the COMSTAT 2 software [43]. All confocal scanning laser microscopy experiments were performed three times, and standard deviations were measured.

### 4.5. Cloning, Expression and Purification of the A. baumannii FtsZ Protein

The full-length (1176 bp) *ftsZ* gene of *A. baumannii* ATCC19606 was amplified from the genomic DNA by PCR using the primers: FtsZSUMOABfor (5′- CAGAGAACAGATTGGTGGTATGGCCTCATTTGAATTTATAGAAG-3′) and FtsZSUMOABrev (5′-ATAAATACCTAAGCTTGTCTTTACTTACGTTGCTGATTTTTCAAG-3′). Primers were designed following the Gibson ^®^ assembly kit instructions (NEB). The PCR product was cloned into the linearized pET-SUMO vector (Invitrogen, Waltham, MA, USA), using the Gibson^®^ assembly kit (New England Biolabs, Ipswich, MA, USA), according to the manufacturer’s instructions. The pET-SUMOFtsZAB vector was transformed into the *E. coli* BL21(DE3) competent cells to express the recombinant protein. After transformation bacteria were grown O/N, 1/50 of the inoculum was inoculated in 3 L of LB, supplemented with kanamycin (50 mg/L), and incubated at 37 °C with shaking until OD600 = 0.6 was reached. The FtsZ expression was induced with 0.5 mM of isopropyl-β-D-thiogalactopyranoside (IPTG, BioChemica, PanReac, AppliChem). The temperature was set at 18 °C and the culture was grown O/N. Cells were collected by centrifugation. The pellet was resuspended in lysis buffer (50 mM Tris-HCl pH 8, 300 mM KCl, 2.5 mM MgCl_2_, 1 mM Dithiothreitol [DTT], 5 mM imidazole and 10% glycerol), supplemented with 1 mM of the nonspecific protease inhibitor phenylmethanesulfonyl fluoride (PMSF, Merck, Readington, NJ, USA), and lysed by sonication. The lysate was centrifuged at 48,000× *g* for 1 hour and the clarified supernatant was loaded on a 1 mL His Trap FF nickel column (1 mL, GE Healthcare, Chicago, IL, USA). The protein was eluted with the lysis buffer with 250 mM imidazole added. The molecular weight of the protein with the SUMO tag was 55.4 kDa. To cleave the SUMO tag from the purified FtsZ, sample was dialyzed O/N at 4 °C, against Buffer A (20 mM Tris-HCl pH 7.8, 100 mM KCl, 2.5 mM MgCl_2_ and 10% glycerol) and the SUMO protease was added to the dialysis. The SUMO tag was removed using a reverse-IMAC. The molecular weight of the protein without the SUMO tag was 42 kDa. The protein was quantified using Qubit Fluorometric Quantification (Thermo Fisher Scientific Inc. Waltham, MA, USA) concentrated to 4 mg/mL and samples were stored at −80 °C.

### 4.6. In Vitro FtsZ GTPase Activity

GTPase activity was assayed at 30 °C using a pyruvate kinase-L-lactic dehydrogenase (PK/LDH) spectrophotometric coupled assay, as previously described in [30], with minor modifications. The reaction mixture contained 50 mM MES (pH 6.5), 5 mM Mg(CH_3_COO)_2_, 100 mM CH3CO2K, 10 U PK/LDH, 0.25 mM NADH, 0.25 mM phosphoenolpyruvate, and 4.8 μM of FtsZ. The assay was initiated by the addition of 1 mM GTP. The experiments were performed in triplicate, and the kinetic constants were determined by fitting the data to the Michaelis–Menten equation using Prism 9. C109 was added in concentrations ranging from 0.5 to 100 μM, and the inhibitory concentration that reduced the enzymatic activity by half (IC_50_) was determined using Prism 9.

### 4.7. In Vitro FtsZ Polymerization Assay

The polymerization of FtsZ was assessed in vitro using a sedimentation protocol, as previously described in [30]. The reaction mixture was set up to contain 50 mM MES (pH 6.5), 5 mM Mg(CH_3_COO)_2_, 100 mM CH_3_CO_2_K, 12 μM SaFtsZ, and 2 mM GTP or GDP. The reaction mixtures were incubated for 10 min at 30 °C and 300 rpm to allow the polymerization to occur. Subsequently, samples were ultracentrifuged at 350,000× *g* for 10 min at 25 °C, and the supernatant was immediately separated from the pellet, which contained the protein polymers. The samples were analyzed by SDS-PAGE on 12% polyacrylamide gels. The in vitro polymerization of FtsZ protein was tested in the presence of increasing concentrations of the compound C109.

### 4.8. Statistical Methods

Analyses were performed using Prism 9.0 (GraphPad). Comparison of more than two groups were performed with the one-way or two-way ANOVA. P values < 0.1 were considered statistically significant (*p* < 0.1, ** *p* < 0.01, *** *p* < 0.001, **** *p* < 0.0001).

## Data Availability

Not applicable.
